# Probiotics as Antioxidant Strategy for Managing Diabetes Mellitus and Its Complications

**DOI:** 10.3390/antiox14070767

**Published:** 2025-06-22

**Authors:** Max Denisson Maurício Viana, Sthefane Silva Santos, Anna Beatriz Oliveira Cruz, Maria Vitória Abreu Cardoso de Jesus, Pedro Santana Sales Lauria, Marvin Paulo Lins, Cristiane Flora Villarreal

**Affiliations:** 1 School of Pharmacy, Federal University of Bahia, Salvador 40170-290, BA, Brazil; max.viana@ufba.br (M.D.M.V.); sthefaness@ufba.br (S.S.S.); annacruz@ufba.br (A.B.O.C.); mariavacj@ufba.br (M.V.A.C.d.J.); pedrosslauria@gmail.com (P.S.S.L.); 2 Department of Anesthesiology, University of California San Diego, San Diego, CA 92037, USA; 3 Faculty of Medicine, Federal University of Mato Grosso, Cuiabá 78060-900, MT, Brazil; marvin.lins@ufmt.br; 4 Brazilian National Institute of Science and Technology on Neuroimmunomodulation, Oswaldo Cruz Institute, Rio de Janeiro 21040-360, RJ, Brazil

**Keywords:** diabetes mellitus, diabetic neuropathy, diabetic nephropathy, oxidative stress, gut microbiota, probiotics, antioxidant, antidiabetic potential

## Abstract

Diabetes mellitus (DM) is a chronic metabolic disorder characterized by impaired glycemic regulation and persistent hyperglycemia, which drives the onset of microvascular complications such as diabetic neuropathy and nephropathy. Chronic hyperglycemia activates oxidative stress pathways and alters gut microbiota composition, both of which contribute to disease progression. In this context, probiotics have emerged as promising therapeutic agents due to their ability to modulate oxidative stress, improve glycemic control, and influence gut microbial balance. This review summarizes preclinical and clinical evidence supporting the antioxidant potential of probiotics in DM management, with a focus on underlying mechanisms. Strains from the *Lactobacillus* and *Bifidobacterium* genera are the most extensively studied and have demonstrated hypoglycemic and antioxidant effects, including the enhancement of key antioxidant enzymes and reductions in lipid peroxidation and nitrosative stress markers. Probiotics have also shown beneficial effects in DM-associated complications, particularly diabetic neuropathy and nephropathy. While clinical data are still limited, recent findings underscore oxidative stress as a critical therapeutic target influenced by probiotic interventions. Overall, current evidence supports probiotics as a complementary strategy for managing DM and its complications, highlighting the need for further well-designed clinical trials exploring diverse strains, formulations, and dosing regimens.

## 1. Introduction

Diabetes mellitus (DM) is a chronic metabolic disorder characterized by sustained hyperglycemia resulting from impaired insulin secretion, insulin action, or both. It includes type 1 (T1D), type 2 (T2D), and gestational diabetes (GDM). T2D accounts for over 90% of cases and is strongly associated with lifestyle factors [1]. The estimates from the 11th edition of the IDF Diabetes Atlas indicate that, in 2024, there were 588.7 million adults (aged 20–79 years) living with diabetes, corresponding to a global prevalence of 11.1%. Projections for 2050 predict a 17% increase in the number of cases, reaching approximately 850.5 million adults worldwide [2].

More than 50% of adults with T2D remain untreated, which increases the risk of severe complications such as diabetic neuropathy (DNR) and nephropathy (DNP), both of which are major microvascular outcomes of chronic hyperglycemia [3]. DNR affects approximately 50% of diabetic patients; it involves peripheral nerve damage, leading to neuropathic pain, sensory loss, and an increased risk of limb amputation [4]. DNP affects 20–40% of individuals with DM and is characterized by proteinuria and progressive renal decline, which contribute significantly to global morbidity and mortality [5].

The pathogenesis of DM involves the complex dysregulation of several biological processes at the cellular level. In the diabetic state, chronic hyperglycemia leads to mitochondrial dysfunction and subsequent excessive production of reactive oxygen species (ROS), which eventually overwhelms antioxidant defenses. Oxidative stress plays a central role in propagating cellular damage and in driving dramatic changes in the composition of the gut microbiota (GM) [6]. Given the critical contribution of oxidative stress to the progression of DM and its complications—including those affecting the gut—new antioxidant agents have emerged as promising therapeutic strategies. Such strategies include the use of probiotics, live microorganisms that confer health benefits to the host when administered at the appropriate doses [7]. By modulating the GM, probiotics may improve glycemic control and mitigate oxidative stress [8].

Clinical and preclinical studies have shown that probiotics modulate factors critical to glycemic regulation, including the lipid peroxidation pathway and biomarkers of oxidative and nitrosative stress [9,10]. A meta-analysis revealed that patients with DNP treated with probiotics showed a significant reduction in malondialdehyde (MDA) levels, along with increases in glutathione (GSH) levels and total antioxidant capacity (TAC) [11]. Although data on DNR remain limited, a recent patent review identified oxidative stress as a central therapeutic target of probiotic innovations [12]. The growing interest in this area is mirrored by the expanding global probiotics market, valued at USD 9 billion in 2023 [13].

Given the key role of oxidative stress in the pathogenesis of DM—whose global prevalence is expected to continue rising—and the growing commercial interest in probiotics due to their antioxidant potential, this review aims to consolidate the current evidence on the use of probiotic strains in managing DM and its major complications, with a particular emphasis on reducing oxidative stress. This review provides a comprehensive overview of emerging microbial strains and their underlying antioxidant mechanisms of action, with the goal of supporting further research into the clinical benefits and applications of probiotics.

## 2. Diabetes Mellitus and Its Major Complications

### 2.1. Etiology and Pathophysiology of Diabetes Mellitus (DM)

The etiology of DM, particularly T2D, is multifactorial and involves a complex interplay between genetic predisposition and environmental factors. Individuals with a family history of DM exhibit a markedly higher risk of developing the condition, suggesting an important hereditary component. However, lifestyle risk factors also play an important role in triggering the disease in genetically susceptible individuals. Relevant risk factors include obesity (especially when there is excessive visceral adiposity), lack of physical activity, and poor eating habits [14]. The combination of hereditary and lifestyle components results in elevated levels of circulating free fatty acids and chronic low-grade inflammation, which frequently leads to insulin resistance.

In the pathophysiology of T2D, peripheral tissues—particularly skeletal muscle and adipose tissue—are affected by insulin resistance. These tissues become less responsive to insulin and their ability to uptake glucose is impaired. In response, pancreatic β-cells increase insulin secretion to maintain euglycemia. Over time, chronic metabolic stress, lipotoxicity, glucotoxicity, and low-grade inflammation contribute to β-cell exhaustion and apoptosis. This increased functional demand placed on pancreatic β-cells eventually leads to their inability to maintain compensatory insulin secretion, resulting in progressive hyperglycemia [15,16]. Concurrently, other hormonal and metabolic changes contribute to the sustained elevation of blood glucose levels; these include hepatic insulin resistance leading to excessive gluconeogenesis, increased glucagon secretion, and altered responses to incretins.

Persistent hyperglycemia contributes to endothelial dysfunction, oxidative stress, and chronic low-grade inflammation [17]. Moreover, hyperglycemia promotes the formation of advanced glycation end-products (AGEs), which activate inflammatory pathways through receptor-mediated mechanisms, further exacerbating tissue damage. Insulin resistance and metabolic dysregulation also enhance the production of ROS, amplifying oxidative stress and promoting cellular dysfunction [18]. Oxidative stress is increasingly recognized as a central driver in the disruption of several metabolic pathways involved in the development of T2D. Excess ROS, generated in response to chronic hyperglycemia and inflammation, can impair insulin signaling, exacerbate pancreatic β-cell damage, and disrupt GM balance [19].

Additional contributors to the pathogenesis of DM include socioeconomic factors that influence access to healthy food and opportunities for physical activity, sleep disturbances, and alterations in the GM [20]. Dysbiosis, or the imbalance of the GM, has been observed in individuals with T2D, who often exhibit reduced microbial diversity and shifts in specific bacterial taxa compared to normoglycemic individuals [21]. These changes may impair glycemic regulation, as resident microbiota play crucial roles in metabolic homeostasis, including modulating inflammation and enhancing antioxidant defenses. Certain microbial metabolites, such as short-chain fatty acids (SCFAs), have been shown to exert insulin-sensitizing and anti-inflammatory effects, suggesting that the composition and functionality of the GM may significantly influence glucose metabolism and DM pathogenesis [22].

Taken together, these interconnected disruptions highlight the complex and systemic nature of DM and its progression to chronic complications [23]. T2D is associated with a range of long-term complications that affect multiple organ systems, including cardiovascular disease, nephropathy, retinopathy, and neuropathy. As DNR and DNP are closely linked to chronic systemic inflammation, oxidative stress, and gut dysbiosis, they represent critical targets for evaluating the use of probiotics as a therapeutic strategy, given that probiotics are known to modulate these pathophysiological processes [24]. Moreover, altered gut microbiota may exacerbate these conditions, reinforcing the rationale for preclinical and clinical research on probiotics as modulators of these core pathophysiological pathways, with the potential to improve the clinical manifestations of DM.

### 2.2. Diabetic Neuropathy (DNR): Causes and Consequences

DNR is one of the most common and distressing complications of DM. It typically develops insidiously, as persistent hyperglycemia leads to microvascular injury that compromises peripheral nerve perfusion, especially in the lower limbs. In addition to chronic hyperglycemia, metabolic abnormalities such as dyslipidemia, low-grade systemic inflammation, and oxidative stress further exacerbate nerve damage [25]. The interplay between mitochondrial dysfunction, lipid peroxidation, nitrosative stress, and impaired antioxidant response accelerates nerve fiber degeneration and hinders axonal regeneration, creating a vicious cycle that progressively worsens over time.

Clinically, DNR presents a broad spectrum of symptoms, including numbness, burning, neuropathic pain, and eventual sensory loss. As sensory fibers deteriorate, patients become prone to undetected injuries, leading to chronic ulcers, infections, and, in severe cases, lower-limb amputations. Neuropathic pain substantially impairs quality of life, underscoring the importance of early detection, optimal glycemic control, and symptomatic management [26]. The involvement of autonomic fibers may also disrupt cardiovascular, gastrointestinal, or genitourinary function, compounding the disease burden [27].

Beyond physical impairments, DNR has profound psychosocial consequences. Patients frequently experience sleep disturbances, depression, anxiety, and a diminished sense of independence, all of which significantly impact their quality of life [28]. Given its widespread effects, DNR demands a multidimensional approach that integrates glycemic control, neuroprotection, and psychosocial support. Without early intervention, progressive nerve damage can severely compromise both functional capacity and psychological resilience.

### 2.3. Kidney Complications in Diabetes: Diabetic Nephropathy (DNP)

DNP is a serious microvascular complication of DM, representing the leading cause of end-stage renal disease worldwide. It arises primarily due to chronic hyperglycemia, which triggers a cascade of harmful events, including glomerular hyperfiltration, thickening of the glomerular basement membrane, mesangial expansion, and, ultimately, progressive glomerulosclerosis [29]. These structural alterations compromise the selective permeability of the glomerulus, leading to protein leakage and a gradual decline in renal function. In parallel, oxidative stress and inflammation, fueled by persistent metabolic imbalance, further damage renal structures, progressively reducing the kidney’s filtration capacity. Moreover, the activation of pro-inflammatory pathways, such as nuclear factor kappa B (NF-κB), perpetuates a chronic inflammatory state within the kidney parenchyma, exacerbating both glomerular and tubular damage [30].

Clinically, patients may initially present with asymptomatic microalbuminuria, which can progress insidiously to overt proteinuria, reduced glomerular filtration rate, hypertension, and fluid retention. Ultimately, untreated DNP culminates in kidney failure and substantially increases cardiovascular risk and overall mortality. At this stage, patients require dialysis or kidney transplantation, drastically reducing their quality of life [5].

Neuropathy and nephropathy are among the most debilitating complications of diabetes, and they share a central pathogenic mechanism—oxidative stress driven by chronic hyperglycemia. This unifying factor not only accelerates tissue damage across organ systems but also creates a pro-inflammatory and pro-oxidative environment that undermines the body’s natural repair mechanisms. By recognizing oxidative stress as a pivotal mediator in the pathophysiology of DM, researchers and clinicians can focus on developing targeted interventions aimed at preserving organ function, slowing disease progression, and improving long-term outcomes. This perspective highlights the therapeutic value of antioxidant strategies—such as probiotic supplementation—that can modulate oxidative pathways and restore physiological balance in affected tissues.

## 3. Mechanisms of Oxidative Damage in Diabetes and Its Complications

As discussed in the previous section, oxidative stress plays a critical role in metabolic diseases like DM. Chronic hyperglycemia promotes excessive ROS production, surpassing the capacity of antioxidant defenses and causing cumulative widespread cellular damage [31], which ultimately leads to major diabetic complications [32]. Abundantly available glucose increases metabolic flux, particularly in insulin-independent tissues, such as nerves and blood vessels. This excess substrate availability overwhelms the electron transport chain, leading to increased ROS production and mitochondrial dysfunction, which is an important early consequence of hyperglycemia. Once mitochondrial integrity has been compromised, a self-perpetuating cycle of damage is established, ultimately leading to vascular and neural complications [33].

Hyperglycemia also triggers other harmful alterations at the cellular level. The reaction of glucose with proteins, lipids, or nucleic acids leads to the formation of advanced glycation end-products (AGEs). Upon interacting with their receptors, AGEs initiate pro-inflammatory and pro-oxidative cascades [34]. The protein kinase C (PKC) pathway is another oxidative amplifier in DM, especially under the influence of elevated diacylglycerol (DAG) levels. Hyperglycemia increases DAG, which in turn activates PKC isoforms, particularly in vascular cells. This leads to impaired blood flow regulation, increased vascular permeability, and the overexpression of inflammatory genes. The oxidative stress triggered by PKC activation is particularly damaging to the kidneys and peripheral nerves, explaining why so many people with poorly controlled DM experience nephropathy or neuropathy as early complications [35].

Altogether, mitochondrial overload, the accumulation of AGEs, and the activation of the PKC pathway constitute a biochemical triad that amplifies oxidative and nitrosative stress in DM. These interrelated mechanisms overwhelm antioxidant defense systems, leading to reduced activity of key enzymes such as superoxide dismutase (SOD), catalase (CAT), GSH, and glutathione peroxidase (GPx), while promoting excessive production of ROS and reactive nitrogen species like nitric oxide (NO). As a result, lipid peroxidation— marked by elevated MDA levels—intensifies cellular injury [30]. Clinically, these processes portend a bad prognosis, highlighting the urgent need for early glycemic control and interventions aimed at restoring redox homeostasis [36].

Antioxidant enzymes such as SOD, CAT, GPx, and GSH play essential roles in mitigating oxidative damage by catalyzing the detoxification of ROS [37]. SOD catalyzes the dismutation of superoxide anions into hydrogen peroxide, which is then broken down by CAT and GPx into water and oxygen, preventing harmful oxidative reactions. GSH acts as a crucial non-enzymatic antioxidant, serving as a substrate for GPx and participating in the regeneration of other antioxidants. In DM, chronic hyperglycemia leads to a marked reduction in the activity and expression of these enzymes, resulting in excessive ROS accumulation [38]. This redox imbalance contributes directly to endothelial dysfunction, mitochondrial damage, and inflammation (key mechanisms in the pathogenesis of both DNR and DNP). In peripheral nerves, oxidative stress impairs axonal transport and myelin integrity, while in the kidneys, it accelerates glomerular injury and tubular apoptosis, exacerbating the progression of renal dysfunction [39,40].

DM complications are also critically influenced by nitrosative stress, characterized by the excessive formation of reactive nitrogen species (RNS). Among these, peroxynitrite (ONOO^−^) is prominently generated through the rapid interaction of nitric oxide (NO) with superoxide anion (O_2_^−^), exerting significant cytotoxic effects [41]. In DNR, excess peroxynitrite contributes to mitochondrial dysfunction and the nitration of cytoskeletal proteins, disrupting axonal transport and contributing to neurodegeneration. It also modifies the ion channels and receptors involved in nociception, intensifying pain signaling pathways [42]. In DNP, nitrosative stress damages podocytes and endothelial cells within the glomeruli, resulting in cytoskeletal disorganization and loss of filtration barrier integrity [43]. Peroxynitrite-induced nitration of key proteins such as manganese superoxide dismutase further impairs renal antioxidant defenses. Additionally, RNS activates fibrotic signaling cascades via transforming growth factor beta and NF-κB pathways, contributing to extracellular matrix accumulation and glomerulosclerosis. These molecular alterations collectively worsen kidney function and accelerate disease progression in diabetic patients [44].

Concurrently, a high concentration of MDA, which serves as a biomarker of lipid peroxidation, is frequently associated with the onset and progression of diabetes-related microvascular complications [45]. MDA is a reactive aldehyde formed as a byproduct of lipid peroxidation, a process in which ROS attack polyunsaturated fatty acids in cell membranes. As a key indicator of oxidative stress, MDA reflects the extent of cellular membrane damage and dysfunction. In DM, chronic hyperglycemia promotes excessive ROS generation, overwhelming antioxidant defenses, and accelerating lipid peroxidation [46]. Elevated MDA levels have been consistently observed in patients with DNR and DNP, suggesting that oxidative damage to neuronal and renal tissues is an important mechanism in the progression of both manifestations [47].

**Figure 1:**
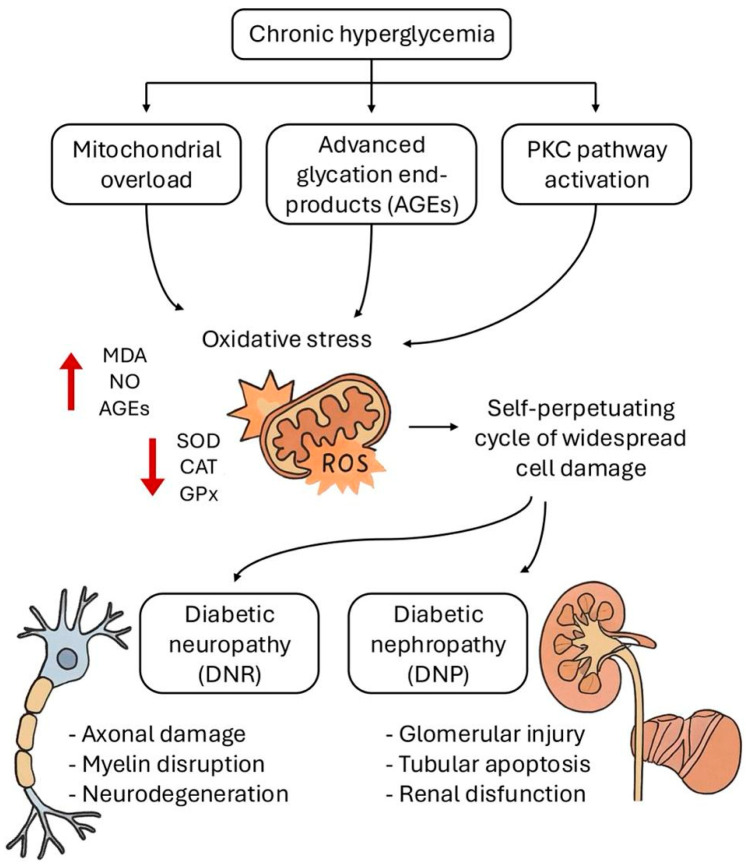
Pathophysiological events leading to oxidative damage in diabetes mellitus and its complications. In diabetes mellitus, chronic hyperglycemia leads to a sustained oxidative imbalance, marked by excessive production of reactive oxygen species (ROS). This environment promotes mitochondrial dysfunction, formation of advanced glycation end products (AGEs), and activation of signaling pathways such as PKC (protein kinase C), contributing to vascular damage. As a result, oxidative and nitrosative stress is amplified, lipid peroxidation increases, evidenced by elevated malondialdehyde (MDA) levels, and endogenous antioxidant defenses are overwhelmed. Key antioxidant factors, including superoxide dismutase (SOD), catalase (CAT), and glutathione peroxidase (GPx) exhibit decreased levels, as well as dysregulation of nitric oxide (NO) levels. Collectively, these alterations accelerate tissue injury and contribute to the progression of complications such as diabetic neuropathy (DNR) and diabetic nephropathy (DNP).

Importantly, oxidative stress in DM extends beyond the nervous and renal systems, compromising the integrity of other organs, such as the liver and gastrointestinal tract. The liver, which plays a central role in glucose and lipid metabolism, undergoes oxidative damage that worsens insulin resistance and contributes to the development of nonalcoholic fatty liver disease—a condition highly prevalent among individuals with T2D [48]. Additionally, the gastrointestinal tract may suffer barrier dysfunction, microbial dysbiosis, and chronic low-grade inflammation, all of which further amplify systemic metabolic dysregulation [49]. Altogether, these biochemical disruptions reinforce the central role of oxidative and nitrosative stress in the development of diabetes-related complications and highlight the need to target redox imbalance as a therapeutic strategy. A summary of the events described in this section is represented in Figure 1.

## 4. Advances in Diabetes Management: From Hypoglycemic Agents to Probiotic Therapeutics

The pharmacotherapy of DM is continuously advancing, with a strong emphasis on achieving and maintaining glycemic control. The effective management of blood glucose levels is essential to mitigate the progression of diabetic complications, as achieving optimal glycemic control prevents further nerve damage and kidney dysfunction. The conventional treatment of DNR should focus primarily on glycemic control, which remains the cornerstone for preventing disease onset and/or slowing its progression [50]. By stabilizing blood glucose levels, the burden of oxidative stress decreases significantly, thereby reducing the self-perpetuating cycle of neural injury. Pharmacological management for symptomatic relief of diabetic neuropathic pain often includes antidepressants or anticonvulsants [51]. Although these drugs do not reverse nerve damage, they improve pain control and quality of life, which can be significant for patients struggling with burning sensations, sleep disturbances, and emotional distress.

Despite the availability of pharmacological options for DNR, conventional therapies often fail to achieve sustained and multidimensional analgesia. They generally do not address the root causes of neuropathic pain, such as chronic inflammation and oxidative stress [52]. Furthermore, adverse effects—such as somnolence, weight gain, psychomotor slowing, and gastrointestinal discomfort—as well as the development of tolerance, can compromise treatment adherence and continuity [53]. Because of these limitations, clinical strategies often combine traditional pharmacological treatment with lifestyle interventions [54].

Strict glycemic control is equally important in the management of DNP, as it helps to reduce the risk of microalbuminuria and slow down the decline of glomerular filtration rate in diabetic patients. Angiotensin-converting enzyme inhibitors and angiotensin II receptor blockers are commonly prescribed to manage hypertension and provide renoprotective effects by reducing glomerular pressure and alleviating proteinuria [55]. Additionally, newer classes of drugs such as sodium–glucose transport protein 2 inhibitors have shown promising results in slowing the progression of DNP by improving renal function and reducing albuminuria, even in patients without overt hyperglycemia [56]. Despite these advances, similarly to DNR, current pharmacological approaches to DNP focus primarily on symptom control and do not reverse the underlying damage caused by chronic hyperglycemia and oxidative stress, both of which continue to drive disease progression in many cases.

Recognizing that both glycemic dysregulation and oxidative stress are central to the pathogenesis of DM and its complications—and that the GM can modulate both—current therapeutic strategies are increasingly shifting away from pancreas-centric models toward gut-focused interventions. This includes targeting incretin hormones such as glucagon-like peptide-1 (GLP-1) and glucose-dependent insulinotropic polypeptide (GIP), which play key roles in glucose homeostasis and insulin secretion [57]. Individuals with DM often exhibit an altered GM profile [58], and this dysbiosis is linked to increased intestinal permeability, systemic inflammation, and impaired glucose metabolism. Accordingly, modulating the GM through probiotic-, prebiotic-, or synbiotic-based therapies is emerging as a promising adjunct—not only for improving metabolic control but also for delaying or preventing the onset of diabetic complications [59,60].

## 5. Antioxidant Potential of Probiotics in Managing Diabetes and Its Complications

**Figure 2:**
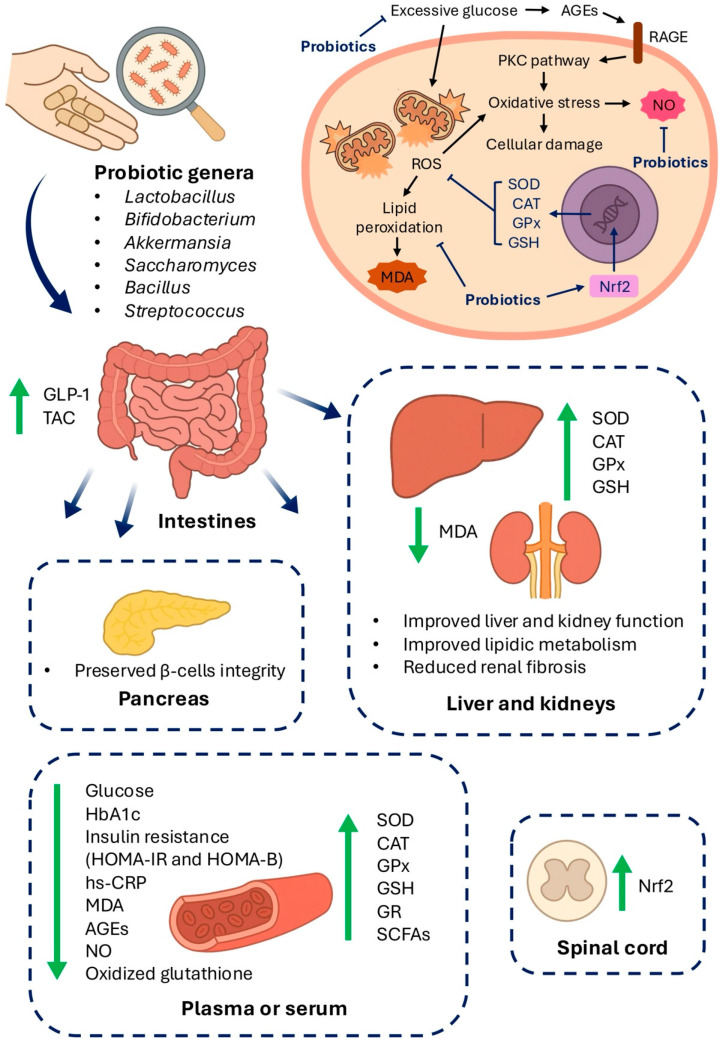
Antioxidant effects and mechanisms of probiotics during diabetes mellitus. Several probiotic strains have been shown to modulate biomarkers of oxidative stress and promote the healthy function of different organs. Combined data from pre-clinical and clinical trials suggest that the antioxidant mechanisms of probiotics are strain-specific and mainly involve the improvement of glycemic control and the induction of enzymes that catalyze the detoxification of reactive oxygen species. Advanced glycation end-products (AGEs); catalase (CAT); glucagon-like peptide-1 (GLP-1); glutathione peroxidase (GPx); glutathione reductase (GR); glutathione (GSH); glycated hemoglobin (HbA1c); homeostasis model assessment of β-cell function (HOMA-B); homeostasis model assessment of insulin resistance (HOMA-IR); high-sensitivity C-reactive protein (hs-CRP); malondialdehyde (MDA); nuclear factor kappa B (NF-κB); nitric oxide (NO); nuclear factor erythroid 2-related factor 2 (Nrf2); protein kinase C (PKC); receptor for advanced glycation end products (RAGE); reactive oxygen species (ROS); short-chain fatty acids (SCFAs); superoxide dismutase (SOD).

Preclinical and clinical evidence suggests that complications arising from chronic hyperglycemia can alter the GM, thereby influencing neural, immunological, endocrine, and redox homeostasis [3]. As a result, probiotics—once primarily associated with gastrointestinal health—have gained attention for their potential benefits in psychiatric, neurodegenerative, and metabolic conditions, including diabetes [61,62]. Given the pivotal role of oxidative stress in the pathophysiology of T2D and its complications, along with the well-documented antioxidant properties of certain probiotic strains, growing interest has emerged around their use as therapeutic agents [12,62]. Key mechanisms by which probiotics may exert beneficial effects include the attenuation of oxidative stress through modulating antioxidant enzyme pathways, the reduction in nitric oxide production, and the inhibition of lipid peroxidation [63]. In the sections that follow, we review the available evidence supporting these mechanisms, highlighting experimental and clinical findings that demonstrate how probiotics may mitigate oxidative damage in T2D. Table 1, Table 2 and Table 3 summarize the main results concerning diabetes and its major complications. Figure 2 illustrates the main antioxidant mechanisms of probiotics during DM and the effects described in the literature for different target organs.

### 5.1. Preclinical Evidence of Antioxidant and Antidiabetic Properties

The investigation of probiotics as therapeutic agents for T2D has gained momentum over the past two decades, particularly in preclinical models that allow for mechanistic insights into glycemic regulation and oxidative stress mitigation. This section highlights key animal studies that demonstrate how specific probiotic strains and formulations exert antioxidant and antidiabetic effects, along with their potential impact on systemic metabolic parameters.

The use of probiotics in the treatment of T2D is a relatively recent approach. Scientific literature on the GM, probiotics, and DM—particularly studies investigating mechanisms of glycemic control—has been emerging since the early 2000s [64]. It has since been reported that dysbiosis may enhance auxiliary microbial functions and promote oxidative stress, highlighting GM alterations as potential predictive biomarkers for DM [65,66]. Given the involvement of GM in organ damage, its modulation appears to be a promising therapeutic strategy for T2D and its complications. A deeper understanding of the pathophysiological processes underlying glycemic homeostasis further supports this approach. Preclinical models of T2D, which replicate key features of the human condition, have enabled the investigation of probiotic-based interventions in this context.

The primary experimental models used to induce diabetes, along with its complications, rely on either chemical induction or genetic manipulation, both of which effectively replicate disease progression and pathophysiological mechanisms, including oxidative stress. Among chemical inducers, alloxan and streptozotocin (STZ) are frequently employed due to their selective accumulation in pancreatic β-cells [67]. Alloxan generates ROS via redox cycling, while STZ exerts cytotoxic effects through DNA alkylation, resulting in β-cell damage [68,69]. Additionally, STZ increases xanthine oxidase activity and NO production, contributing to oxidative and nitrosative stress. STZ can be administered alone or in combination with other interventions, such as a high-fat diet (HFD) or nicotinamide. The STZ/HFD and STZ/nicotinamide models were developed to mimic the transition from pre-diabetes or insulin resistance to overt T2D, as they induce gradual β-cell apoptosis—in the latter case, modulated by the partial protective effect of nicotinamide [70,71]. These models are extensively used to evaluate therapeutic strategies, including the effects of probiotics.

Zeng et al. [72] evaluated the effects of *Lactobacillus paracasei* L14 supplementation (10^10^ colony-forming units, CFU) over 12 weeks in HFD/STZ-induced diabetic rats. The strain significantly reduced hyperglycemia preserved pancreatic β-cells integrity, and improved liver function. It also enhanced antioxidant defense by markedly increasing serum levels of CAT, GPx, and SOD, while reducing MDA to near basal levels. Additionally, the strain modulated GM composition, attenuating intestinal dysbiosis and suggesting systemic regulatory effects. In addition, a genomic analysis of the strain identified 16 genes related to the expression of antioxidant factors and 20 genes related to the synthesis of exopolysaccharides, which are microbial metabolites related to bacterial adhesion, aggregation and survival, i.e., fundamental properties for a probiotic strain.

A similar strain, *L. paracasei* NL41 (10^10^ CFU), was tested in the same model. After 12 weeks of treatment, it significantly enhanced the activity of the antioxidant enzymes GPx, SOD, and CAT, restoring these enzymes to levels observed in the healthy controls. Concurrently, MDA levels were reduced, indicating decreased oxidative damage. Notably, significant reductions in glycemic levels, HbA1c, and insulin resistance were reported and associated with the inhibitory activity of dipeptidyl peptidase 4 and α-glucosidase enzymes in previous studies of the group with the strain. Immunofluorescence analysis further revealed that NL41 treatment mitigated β-cell damage in pancreatic islets, suggesting a protective effect on endocrine function [73].

Comparable antioxidant and antidiabetic properties were observed with another *Lactobacillus* species. In alloxan-induced diabetic mice, the administration of *L. agilis* for 15 days significantly reduced glycemia and enhanced antioxidant enzyme activities (SOD, CAT, and GSH) compared to the vehicle-treated controls. Additionally, the strain showed anti-inflammatory potential [74].

In a STZ + nicotinamide model, Pegah et al. [75] investigated a multistrain formulation containing seven *Lactobacillus* and *Bifidobacterium* species (5 × 10^10^ CFU)—administered alone or in combination with resveratrol—in Wistar rats for 4 weeks. Probiotic supplementation alone significantly reduced the insulin resistance index—an effect associated with increased GLP-1 levels. Moreover, TAC was significantly elevated in the probiotic group, but not in the resveratrol-only group, when compared to diabetic controls. However, a reduction in total oxidative status was only observed in the group that received the combined treatment, suggesting synergistic antioxidant effects when probiotics were co-administered with resveratrol.

Another multistrain formulation, Probioglu^TM^, comprising *L. salivarius* subsp. *salicinius* AP-32, *L. johnsonii* MH-68, *L. reuteri* GL-104, and *B. animalis* subsp. *lactis* CP-9, was evaluated in STZ + nicotinamide-induced diabetic rats, also subjected to HFD. Over an 8-week period, Probioglu^TM^ significantly improved glycemic and lipid profiles, attenuated STZ-induced β-cell death, increased serum antioxidant markers and SOD activity, and decreased serum MDA. Among all groups, the 10 × Probioglu^TM^ treatment showed the most pronounced effects. Additionally, the authors determined the presence of SCFAs (acetic, propionic, and butyric acids) in the supernatant of individual probiotics strains cultured in vitro using high-performance liquid chromatography. These SCFAs were positively correlated with improved glycemic control [76].

*Akkermansia muciniphila* has also emerged as a promising probiotic for metabolic disorders. The oral administration of *A. muciniphila* (DSM 22959) (5 × 10^6^–10^8^ CFU) for 4 weeks in STZ- and HFD-induced diabetic rats reduced glucotoxicity, alleviated oxidative stress, and restored GM diversity, reinforcing its proposed benefits for metabolic health [77].

Song et al. [78] assessed the preventive effects of several strains—*L. plantarum* CKCC1312, *L. gasseri* CKCC1913, and two *L. fermentum* strains (CKCC1858 and CKCC1369)—in STZ + HFD-induced diabetes model in mice. Most strains improved glucose metabolism through GLP-1 modulation, the normalization of fasting glucose and insulin levels, the upregulation of glucose transporters, improved insulin sensitivity, and the preservation of pancreatic structure and function. Antioxidant effects included significant increases in hepatic SOD, CAT, and GSH, and reductions in MDA. Genomic sequencing was performed on mouse feces to analyze the microbial profile and related genes. Genes related to 17 metabolic pathways—including glycometabolism and lipid metabolism—were found. The CKCC1312 strain presented the best microbial profile, with a greater abundance of species from the genera *Lactobacillus* and *Akkermansia*, recognized for their metabolic therapeutic properties.

In another alloxan-induced model, Kumar et al. [79] investigated the effects of *L. fermentum* RS-2 (10^8^ CFU), administered via fermented milk for over 60 days. The strain significantly boosted antioxidant enzyme activity (SOD, CAT, and GPx) in serum and liver samples, reduced fasting glucose, and attenuated disease severity. The study also performed *Lactobacillus* counts every 7 days in fecal samples. Interestingly, the group treated with the RS-2 strain showed stable *Lactobacillus* counts throughout the experimental period, while the diabetic control group showed progressive loss in counts, corroborating the findings of dysbiosis induced by the condition. The authors emphasized the need for clinical validation to support its potential use in functional dairy products.

Preclinical studies have also shown benefits from synbiotic formulations, which are combinations of probiotics and prebiotics. In an alloxan-induced diabetes model, a synbiotic containing *L. casei* (1 × 10^7^ CFU/mL) and bioactive extracts of *Cleome droserifolia* was administered in rats for 30 days. The probiotic alone and the synbiotic mixture showed comparable results; both reduced MDA levels and increased SOD and CAT activities, mitigating oxidative damage to β-cells. Additionally, improvements were observed in glycemia, lipid profiles, and liver and kidney function [80].

Collectively, preclinical evidence supports the antioxidant and antidiabetic potential of probiotic and synbiotic interventions across various animal models of DM (Table 1). Probiotic strains from the genera *Lactobacillus*, *Bifidobacterium*, and *Akkermansia*—administered at doses ranging from 10^6^ to 10^10^ CFU—led to reductions in oxidative stress markers (e.g., MDA) and enhanced antioxidant defenses (SOD, CAT, GPx, GSH). Improvements in glycemic control, insulin sensitivity, and the preservation of pancreatic β-cell integrity were consistently reported. Additional benefits—such as anti-inflammatory effects, improved lipid profile, and enhanced liver and kidney function—underscore the therapeutic potential of microbiota-targeted therapies in managing diabetes and its complications.

**Table 1:** Preclinical evidence supporting antidiabetic and antioxidant effects of probiotic supplementation.

Strains	Experimental Models	Key Findings	Reference
*L. paracasei* L14	High-fat diet (HFD)/Streptozotocin (STZ)-induced diabetic rats	↓ Blood glucose, preserved pancreatic β-cells integrity, improved liver function, ↓ serum malondialdehyde (MDA) levels, ↑ serum superoxide dismutase (SOD), catalase (CAT), and glutathione peroxidase (GPx) levels.	[72]
*L. paracasei* NL41	HFD/STZ-induced diabetic rats	↓ Blood glucose, ↓ Insulin resistance, ↓ glycated hemoglobin, preserved pancreatic β-cells integrity, ↓ liver MDA levels, ↑ liver SOD, CAT, and GPx levels.	[73]
*L. agilis*	Alloxan-induced diabetic mice	↓ Blood glucose, ↑ serum SOD, CAT, and reduced glutathione (GSH) levels, anti-inflammatory activity.	[74]
*L. plantarum*, *L. bulgaricu*, *L. casei*, *B. infantis*, *L. acidophilus*, *B. longum*, *B. breve* + resveratrol	STZ + nicotinamide-induced diabetic rats	Probiotic alone or synbiotic: ↓ Insulin resistance, ↑ glucagon-like peptide-1 (GLP-1), and ↑ total antioxidant capacity in gut tissue. Synbiotic: ↓ Total oxidative status in gut tissue.	[75]
*L. salivarius* subsp. *salicinius* AP-32, *L. johnsonii* MH-68, *L. reuteri* GL-104, and *B. animalis* subsp. *lactis* CP-9 (Probioglu^TM^)	HFD/STZ + nicotinamide-induced diabetic rats	↓ Blood glucose, ↓ insulin levels, ↓ insulin resistance, ↓ homeostatic model assessment of insulin resistance (HOMA-IR), preserved pancreatic β-cells integrity, ↓ serum MDA levels, and ↑ serum SOD levels.	[76]
*Akkermansia muciniphila* (DSM 22959)	HFD/STZ-induced diabetic rats	↓ Blood glucose and serum MDA levels.	[77]
*L. plantarum* CKCC1312, *L. gasseri* CKCC1913, *L. fermentum* CKCC1858, and *L. fermentum* CKCC1369	HFD/STZ-induced diabetic mice	↓ Blood glucose, ↓ insulin levels, ↓ insulin resistance, ↓ HOMA-IR, ↑ GLP-1, preserved pancreatic β-cells integrity and function, ↓ liver MDA levels, ↑ liver SOD, CAT, and GSH levels, improved lipidic metabolism, anti-inflammatory activity.	[78]
*L. fermentum* RS-2	Alloxan-induced diabetic rats	↓ Blood glucose, ↑ blood and liver SOD, CAT, and GPx levels, improved lipidic metabolism.	[79]
*L. casei* + bioactive extracts of *Cleome droserifolia*	Alloxan-induced diabetic rats	↓ Blood glucose and ↓ liver and kidney MDA levels, ↑ liver and kidney SOD, and CAT levels, improved lipidic metabolism, improved kidney and liver functions.	[80]

Data from preclinical diabetes models, underscoring key outcomes on oxidative stress and glycemic control following probiotic and synbiotic interventions. Arrows indicate direction of change observed after intervention: ↑ increase, ↓ decrease. CAT—catalase, GLP-1—glucagon-like peptide-1, GPx—glutathione peroxidase, GSH—reduced glutathione, HFD—high-fat diet, HOMA-IR—homeostatic model assessment of insulin resistance, MDA—malondialdehyde, SOD—superoxide dismutase, STZ—streptozotocin.

### 5.2. Clinical Evidence of the Antidiabetic Potential of Probiotics

Beyond preclinical studies, substantial clinical evidence supports the antioxidant properties and therapeutic efficacy of probiotic strains in patients with DM. A systematic review and meta-analysis included 16 randomized clinical trials and a total of 1060 patients with DM. Most studies evaluated probiotic supplementation, while four investigated synbiotic formulations. The probiotics used comprised inulin and isomalt, administered in capsules, bread, yogurt, honey, and milk. The results suggested that probiotic and synbiotic supplementation may be useful in controlling glycemic homeostasis, as well as modulating biomarkers of oxidative stress (MDA, GSH, NO, and TAC) in diabetic patients, as demonstrated in at least 10 of the 16 trials included in the analysis [81]. Other reports corroborate these findings.

Probiotic formulations have been proposed, with yogurt serving as the most common delivery vehicle. In a randomized, double-blind, controlled clinical trial involving 64 patients with T2D, the effects of probiotic yogurt containing *Lactobacillus acidophilus* LA5 and *Bifidobacterium lactis* B12 (10^6^ CFU/g) were assessed over a 6-week intervention. Compared to conventional yogurt, the probiotic formulation significantly reduced fasting blood glucose and HbA1c levels. Moreover, serum activities of SOD and GPx, as well as TAC, were markedly increased relative to baseline. These findings support the use of probiotic-based dietary strategies for improving glycemic control and mitigating oxidative stress in T2D patients [82].

Diabetic patients are predisposed to vascular complications, including coronary arterial disease. In a clinical study, the effects of a 12-week intervention with probiotic capsules containing *Lactobacillus* and *Bifidobacterium* species (2 × 10^6^ CFU) were evaluated in patients with T2D and comorbid coronary arterial disease. The intervention improved glucose metabolism, as evidenced by reductions in fasting blood glucose and serum insulin levels. Regarding oxidative stress, key biomarkers were favorably modulated with decreases in hs-CRP and increases in TAC and GSH levels. The effects were associated with an increase in SCFA in the colon and gene expression of the enzyme glutamate-cysteine ligase, a catalyst for GSH synthesis. These outcomes suggest that probiotic supplementation may contribute to improved glycemic control, reduced oxidative stress, and the modulation of lipid profiles, collectively lowering cardiovascular risk in T2D patients [83].

Another study also administered a combination of *Lactobacillus* and *Bifidobacterium* species (2 × 10^9^ CFU/g) in T2D patients. Following 12 weeks of probiotic supplementation, significant improvements were observed in parameters related to glucose metabolism and lipid profile. Moreover, a reduction in plasma MDA levels and an increase in TAC were observed. The authors discussed that the significant effects of probiotic use were related to the production of SCFAs, such as butyrate, in the colon. Notably, the intervention also led to a significant decrease in ulcer length, underscoring additional therapeutic benefits of probiotic use in diabetic individuals [84].

A clinical trial involving 136 diabetic patients was conducted by Mirmiranpour et al. [85]. The study evaluated the effects of *L. acidophilus* PTCC1643 administered either as a standalone probiotic or co-encapsulated with cinnamon (*Cinnamomum zeylanicum*) in a synbiotic formulation. After three months of intervention, both approaches reduced fasting blood glucose and glycated hemoglobin compared to the diabetic group. Interestingly, the administration of the isolated probiotic strain (10^8^ CFU)—but not the synbiotic—resulted in the enhanced activity of SOD, CAT, and GPx, suggesting distinctive metabolic benefits. However, only the synbiotic formulation effectively reduced AGE levels. The species was associated with reduced blood glucose levels because these bacteria produce lactic acid. High amounts of lactic acid in the epithelium reduce glucose uptake by the intestine. These findings highlight the therapeutic potential of the strain, whether administered alone or in combination with bioactive compounds, in managing DM.

Another synbiotic formulation, comprising seven probiotic strains combined with 100 mg of fructooligosaccharides, was evaluated in a double-blind, placebo-controlled clinical trial involving 54 patients with T2D. Following eight weeks of intervention, synbiotic supplementation demonstrated a significant attenuation in the rise of fasting blood glucose levels, along with reductions in systemic inflammatory markers and hs-CRP, and increased total plasma GSH, compared to the placebo group. The authors posited that the effects possibly resulted from increased SCFA production [86].

The trend of testing synbiotics with antidiabetic and antioxidant properties is becoming quite common. The therapeutic effects of a synbiotic food product enriched with *L. sporogenes* (10^7^ CFU), inulin, and beta-carotene were investigated in a randomized, double-blind, placebo-controlled clinical trial involving 51 patients with T2D over a period of 6 weeks. In the synbiotic-supplemented group, significant improvements were observed in the plasma insulin levels, as well as the HOMA-IR, HOMA-B, NO, and GSH concentrations [87]. The authors argue that the antioxidant properties of this strain are related to the production of SCFA (butyrate), which drives GSH synthesis by providing NADPH. It is important to emphasize that the effects of the isolated strain or at higher doses—parameters that have been shown to be influential in other studies—have not yet been investigated.

A recent, randomized, placebo-controlled clinical trial assessed the effects of a synbiotic containing *Bifidobacterium animalis* (5 × 10^10^ CFU), administered either alone or in combination with galacto-oligosaccharides, on glycemic control and oxidative stress parameters. Following 12 weeks, only the synbiotic group exhibited a significant reduction in the HbA1c, HOMA-IR, and insulin levels. In terms of oxidative stress, GSH levels increased significantly in both probiotic and synbiotic groups, but only the synbiotic group showed higher levels compared to the placebo. A reduction in ROS was also observed exclusively in the synbiotic group. The overall improvement in T2D parameters was associated with GLP-1 secretion by the mixture. These results highlight the superior effect of the synbiotic formulation in modulating oxidative stress and improving glycemic homeostasis [88].

The role of probiotic supplementation in GDM, particularly concerning its antioxidant properties, remains poorly explored. A systematic review and meta-analysis showed that probiotic administration in women with GDM was associated with significant reductions in the fasting blood glucose, hs-CRP, and MDA levels. However, no significant effects were observed on the GSH or NO levels. The meta-analysis included seven randomized clinical trials involving a total of 462 women with GDM, all of which are considered high-quality studies. The mean duration of probiotic intervention across the included studies was ≤6 weeks, with administered doses generally below 6 × 10^9^ CFU predominantly using multistrain probiotic formulations. While the overall evidence indicates modest efficacy, the findings underscore the need for further research exploring alternative strains and/or higher dosages to optimize therapeutic outcomes in this population [89].

In this context, Hajifaraji et al. [90] investigated the effects of a multistrain probiotic formulation (>4 × 10^9^ CFU), containing *L. acidophilus* LA-5, *Bifidobacterium* BB-12, *Streptococcus thermophilus* STY-31, and *L. delbrueckii bulgaricus* LBY-27 administered to women with GDM. Following a 4-week intervention period, the randomized clinical trial demonstrated a significant reduction in inflammatory biomarkers, such as hs-CRP and tumor necrosis factor alpha. Notably, the probiotic mixture decreased all oxidative stress markers evaluated, suggesting a potential anti-inflammatory and antioxidant effect of probiotics in GDM management.

Therefore, complementing the promising results observed in animal models, clinical investigations have further explored the use of probiotic and synbiotic formulations in the treatment of T2D and GDM (Table 2). The main genera tested were also *Lactobacillus* and *Bifidobacterium*—administered at doses ranging from 10^6^ to 10^10^ CFU—over 4 to 12 weeks. These probiotic strains or synbiotic combinations were delivered through yogurt or capsules. These approaches led to notable improvements in glycemic indices and favorable changes in oxidative stress markers. Some interventions also exerted anti-inflammatory effects and improved lipid metabolism. The overall trend highlights the clinical value of microbiota-targeted therapies.

To date, both preclinical and clinical evidence consistently support the antioxidant and glycemic-regulating effects of various probiotic strains. Despite the growing body of literature, most studies underscore the need for further investigations in diabetic populations, particularly involving diverse strains and dosing regimens, to inform the development of evidence-based clinical practice guidelines. Moreover, given the demonstrated therapeutic potential of specific probiotic strains in GDM and T2D, the present review reinforces the importance of future research targeting the management of these primary clinical manifestations.

**Table 2:** Clinical evidence supporting antidiabetic and antioxidant effects of probiotic supplementation.

Strains	Experimental Models	Key Findings	Reference
*L. acidophilus* LA5 and *B. lactis* B12	Patients with type 2 diabetes (T2D)	↓ Blood glucose, ↓ glycated hemoglobin (HbA1c), ↑ serum superoxide dismutase (SOD), and glutathione peroxidase (GPx) levels, and ↑ total antioxidant capacity (TAC).	[82]
*B. bifidum*, *L. casei*, and *L. acidophilus*	Patients with T2D	↓ Blood glucose, ↓ Insulin levels, ↓ serum high-sensitivity C-reactive protein (hs-CRP), ↑ reduced glutathione (GSH) levels, and ↑ serum TAC, improved lipidic metabolism.	[83]
*L. acidophilus*, *L. casei*, *L. fermentum*, and *B. bifidum*	Patients with T2D	↓ Blood glucose, ↓ Insulin levels, ↓ HbA1c, ↓ serum hs-CRP, ↓ plasmatic malondialdehyde (MDA) levels, ↑ plasmatic TAC, and ↓ ulcer length, improved lipidic metabolism.	[84]
*L. acidophilus* PTCC1643 + cinnamon	Patients with T2D	Probiotic alone or synbiotic: ↓ Blood glucose and HbA1c. Probiotic alone: ↑ serum SOD, catalase, and GPx levels. Synbiotic: ↓ serum advanced glycation end products levels.	[85]
*L. acidophilus*, *L. casei*, *L. rhamnosus*, *L. bulgaricus*, *B. breve*, *B. longum*, *S. thermophilus* + fructooligosaccharides	Patients with T2D	↓ Blood glucose, ↓ serum hs-CRP, and ↑ plasmatic GSH levels.	[86]
*L. sporogenes* + inulin, and beta-carotene	Patients with T2D	↓ Insulin levels, ↓ homeostatic model assessment of insulin resistance (HOMA-IR), ↓ homeostasis model assessment of β-cell function, ↓ plasmatic nitric oxide and ↓ GSH levels, improved lipidic metabolism.	[87]
*Bifidobacterium animalis* + galacto-oligosaccharides	Patients with T2D	↓ Blood glucose, ↓ Insulin levels, ↓ HOMA-IR, ↓ HbA1c, ↑ glucagon-like peptide-1, and ↑ serum GSH levels, and anti-inflammatory activity.	[88]
*L. acidophilus* LA-5, *Bifidobacterium* BB-12, *Streptococcus thermophilus* STY-31, and *L. delbrueckii bulgaricus* LBY-27	Patients with gestational diabetes mellitus	↓ Blood glucose, ↓ serum hs-CRP, and ↓ MDA levels, and anti-inflammatory activity.	[90]

Data from clinical trials, underscoring key outcomes on oxidative stress and glycemic control following probiotic and synbiotic interventions. Arrows indicate direction of change observed after intervention: ↑ increase, ↓ decrease. GPx—glutathione peroxidase, GSH—reduced glutathione, HbA1c—glycated hemoglobin, HOMA-IR—homeostatic model assessment of insulin resistance, hs-CRP—high-sensitivity C-reactive protein, MDA—malondialdehyde, SOD—superoxide dismutase, T2D—type 2 diabetes, TAC—total antioxidant capacity.

### 5.3. Therapeutic Potential in DNR

As previously discussed, oxidative stress plays a significant role in DNR and associated pain. These events, along with neuroinflammation, sensitization, and plasticity within pain pathways, likely sustain sensory DNR even after glycemic normalization. Combined with dysbiosis, these processes may further exacerbate or perpetuate chronic pain. Thus, probiotics may also exert therapeutic effects on DM complications, such as DNR, through mechanisms independent of glycemic control, given their antioxidant, anti-inflammatory, neuromodulatory, and GM-restoring potential. Although there is no clinical evidence supporting probiotics as a therapeutic strategy for DNR, recent preclinical studies have emerged to elucidate their potential role in this context. It is a fact that the glycemic-regulating and antioxidant activities of several probiotic strains are well established. However, few studies have explored these properties in managing DNR.

Shabani et al. [59] evaluated the effects of a 21-day treatment with a probiotic mixture containing *Lactobacillus* and *Bifidobacterium* species (10^9^ CFU/strain) in a murine model of diabetic neuropathy. The probiotic formulation significantly attenuated the sensory manifestations associated with DNR, reduced the serum MDA levels, and increased the enzymatic activity of serum SOD and GPx, demonstrating the antioxidant activity of the strain mixture and its potential to modulate neuropathic pain. Notably, the observed analgesic and antioxidant effects were not dependent on glycemic control, as blood glucose levels were not significantly altered.

Recently, it has been demonstrated that the intake of *L. acidophilus* LA85 may confer benefits in glycemic control and pain management in an experimental model of DNR. STZ-induced diabetic mice received LA85 at doses of 1.0 × 10^7^ or 1.0 × 10^9^ CFU. Both dosages effectively reduced blood glucose levels; however, the higher dose exhibited a more pronounced attenuation of the behavioral signs of neuropathic pain. This was accompanied by significant reductions in MDA and nitrite amounts, as well as increased activity of SOD, CAT, and GPx, along with the upregulation of Nrf2 in the spinal cord of diabetic mice. The authors reported that the long-lasting antinociceptive effect induced by LA85 during DNR may be associated with the reestablishment of redox homeostasis in the spinal cord [63].

Although studies specifically investigating the role of probiotics in DNR remain limited, there is a substantial and growing body of evidence supporting their efficacy in managing DM. Thus, exploring probiotic interventions in the broader context of DM is relevant and scientifically justified. The results presented in this review reinforce the therapeutic potential of probiotics and strongly advocate the need for further research focused on their application in the prevention and treatment of NDR and other manifestations, such as NDP.

### 5.4. Evidence in Diabetic Nephropathy (DNP)

DNP is a complication of DM that develops primarily as a result of chronic hyperglycemia, which triggers a cascade of pathological processes, including increased oxidative stress and systemic inflammation. Recent studies have expanded our understanding of how probiotics exert renoprotective effects, particularly by modulating oxidative stress via the gut–kidney axis and improving glycemic control [91,92].

Kuo et al. [93] conducted a preclinical study using db/db mice, a model characterized by severe and persistent hyperglycemia due to a mutation in the leptin receptor gene. Mice were treated with a probiotic formulation containing *L. acidophilus* TYCA06, *B. longum* BLI-02, and *B. bifidum* VDD088 for 8 weeks, at doses of 1.025 × 10^9^ or 5.125 × 10^9^ CFU/kg/day. The treatment improved renal function, glycemic control, and attenuated renal fibrosis. To assess the antioxidant potential of strains, in vitro DPPH (2,2-diphenyl-1-picrylhydrazyl) assays were conducted. Among them, *B. longum* BLI-02 (2 × 10^9^ CFU) demonstrated the highest efficacy, enhancing antioxidant activity by 57.1%, as indicated by DPPH reduction. *L. acidophilus* TYCA06 and *B. longum* BLI-02 significantly increased acetic acid levels, suggesting SCFA-linked therapeutic effects.

In the same year, Sun et al. [94] utilized an STZ-induced diabetic kidney disease model in an outbred mouse strain and administered *Lactiplantibacillus plantarum* NKK20 (1 × 10^7^ CFU) for nine weeks. The probiotic elevated fecal butyrate levels, enhanced SOD activity, and reduced MDA and serum AGEs, while markedly attenuating renal fibrosis. These effects were attributed to butyrate’s ability to modulate oxidative stress and inflammatory responses. Complementary in vitro experiments in HK-2 cells stimulated with AGEs showed that butyrate improved cellular integrity, reduced fibrotic markers, and suppressed PI3K/Akt signaling, a key pathway in oxidative stress and profibrotic signaling, supporting the observed renoprotective effects of NKK20.

Abreu et al. [95] investigated the effects of *Saccharomyces boulardii* in an STZ-induced diabetes model in mice treated for 8 weeks at 5 × 10^7^ CFU. The authors reported significant increase in the enzymatic activity of key antioxidant markers, including SOD, CAT, and GPx in the kidney, suggesting *S. boulardii* may support the kidney’s antioxidant defenses and help mitigate diabetes-related damage. These benefits were associated with the positive regulation of dopaminergic and serotonergic levels in kidney tissue. Both are closely related to renal homeostasis by increasing renal blood flow and glomerular filtration rate, in addition to reducing inflammation and renal fibrosis. This finding was accompanied by favorable shifts in GM composition, including increased *Firmicutes* and *Bacteroidetes* and reduced *Proteobacteria*, *Verrucomicrobia*, *Bacteroides*, and *Akkermansia*, which may indeed influence neurotransmitter production and oxidative balance.

Although clinical studies remain limited, early findings support the potential role of probiotics in DNP management. Miraghajani et al. [96] conducted a randomized controlled trial on 48 patients with DNP, administering 200 mL/day of soy milk containing *L. plantarum* A7 (2 × 10^7^ CFU/mL) or plain soy milk for 8 weeks. The probiotic group showed increased serum GSH and enhanced GPx and glutathione reductase activity. No significant changes were seen in serum MDA or TAC, suggesting the selective enhancement of antioxidant defenses. Although the exact mechanisms are unknown, it is proposed that *L. plantarum* A7 may help reduce the production of ROS, enhance antioxidant defenses, and restore the GSH/GSSG balance.

Similarly, Soleimani et al. [97] studied 60 diabetic patients on hemodialysis receiving a probiotic capsule with *L. acidophilus*, *L. casei*, and *B. bifidum* (2 × 10^9^ CFU/g each) for 12 weeks. Probiotic supplementation improved glycemic indices (fasting glucose, insulin, HOMA-IR, HbA1c) and reduced serum hs-CRP and MDA, indicating improved systemic inflammation and oxidative stress control. The authors suggest that these effects may be related to enhanced SCFA production, reduced oxidative radical formation, and improved antioxidant-related enzyme expression.

A placebo-controlled clinical trial [98] further assessed the impact of a probiotic mixture (*L. acidophilus* ZT-L1, *B. bifidum* ZT-B1, *L. reuteri* ZT-Lre, *L. fermentum* ZT-L3; 2 × 10^9^ CFU/each) in 60 DNP patients over 12 weeks. Supplemented individuals showed reductions in fasting glucose, insulin, HOMA-IR, hs-CRP, MDA, and AGEs, with increased serum GSH and improved lipid profiles, confirming both metabolic and antioxidant benefits. These effects were also related to the action of SCFAs produced by probiotics. However, gut microbiota composition was not assessed, which should be considered when interpreting the results.

In 2018, Mazruei-Arani et al. [99] tested probiotic honey containing *Bacillus coagulans* T11 IBRC-M10791 (10^8^ CFU) against conventional honey in 60 DNP patients over 12 weeks. The probiotic group showed improved insulin metabolism, lipid profiles, renal function markers, and reduced hs-CRP and MDA levels. Other oxidative stress parameters (NO, TAC, and GSH) remained unchanged, suggesting strain- and matrix-specific effects of *B. coagulans*.

Both preclinical and clinical studies consistently support the antioxidant properties and metabolic benefits of probiotic supplementation in managing DNP. While clinical trials specifically targeting this complication of DM remain relatively few, the available data point toward a promising adjuvant therapy for managing DNP. These benefits appear to arise from both local gut–kidney axis modulation and systemic antioxidant and anti-inflammatory mechanisms. Further well-designed, large-scale clinical trials involving diverse strains and optimized dosing regimens are warranted to validate and expand these findings.

**Table 3:** Overview of preclinical and clinical evidence on probiotic effects in diabetic neuropathy (DNR) and nephropathy (DNP).

Strains	Complication	Models/Disease	Key Findings	Reference
*Lactobacillus* spp. + *Bifidobacterium* spp. mixture	Diabetic neuropathy (DNR)	Streptozotocin (STZ)-induced diabetic rats	↓ serum malondialdehyde (MDA) levels, ↑ serum superoxide dismutase (SOD) and glutathione peroxidase (GPx) levels, and attenuates sensory manifestations associated with DNR.	[59]
*L. acidophilus* LA85	DNR	STZ-induced diabetic mice	↓ Blood glucose, ↓ MDA and nitric oxide levels, ↑ SOD, catalase (CAT), and GPx levels, ↑ nuclear factor erythroid 2-related factor 2 in the spinal cord, and attenuates sensory manifestations associated with DNR.	[63]
*L. acidophilus* TYCA06, *B. bifidum* VDD088, and *B. longum* BLI-02	Diabetic nephropathy (DNP)	db/db mice 2,2-diphenyl-1-picrylhydrazyl (DPPH) assay	↓ Blood glucose, improved renal function, and ↓ renal fibrosis. *B. longum* increased antioxidative activity in the DPPH assay.	[93]
*L. plantarum* NKK20	DNP	STZ-induced diabetic ICR mice HK-2 cell culture	↓ Blood glucose, ↑ serum butyrate and SOD levels, ↓ serum MDA and advanced glycation end products (AGEs) levels, and anti-inflammatory activity. Inhibition of renal injury and fibrosis.	[94]
*S. boulardii* THT 500101	DNP	STZ-induced diabetic mice (DNP)	↓ Blood glucose, ↑ SOD, CAT and GPx levels in kidney tissue, and improved renal function.	[95]
*L. plantarum* A7	DNP	Patients with DNP	↑ serum reduced glutathione (GSH), GPx and glutathione reductase levels, ↓ serum oxidized glutathione levels, and selective enhancement of antioxidant defenses.	[96]
*L. acidophilus*, *L. casei*, and *B. bifidum*	DNP	Diabetic patients on hemodialysis	↓ Blood glucose, ↓ homeostatic model assessment of insulin resistance (HOMA-IR), ↓ glycated hemoglobin, ↓ serum MDA and high-sensitivity C-reactive protein (hs-CRP) levels.	[97]
*L. acidophilus* ZT-L1, *L. reuteri* ZT-Lre, *L. fermentum* ZT-L3, and *B. bifidum* ZT-B1	DNP	Patients with DNP	↓ Blood glucose, ↓ HOMA-IR, ↓ plasma MDA, serum hs-CRP and AGEs levels, ↑ serum GSH levels, and improved lipid profile.	[98]
*B. coagulans* T11 IBRC-M10791	DNP	Patients with DNP	↓ HOMA-IR, ↓ insulin levels, ↓ plasma MDA, and serum hs-CRP levels, and improved lipid profile.	[99]

Findings from preclinical diabetes models and clinical trials, emphasizing key outcomes related to oxidative stress and DM-associated complications following probiotic interventions. Arrows indicate direction of change observed after intervention: ↑ increase, ↓ decrease. AGEs—advanced glycation end products, CAT—catalase, DNR—diabetic neuropathy, DNP—diabetic nephropathy, DPPH—2,2-diphenyl-1-picrylhydrazyl, GPx—glutathione peroxidase, GSH—reduced glutathione, HOMA-IR—homeostatic model assessment of insulin resistance, hs-CRP—high-sensitivity C-reactive protein, MDA—malondialdehyde, SOD—superoxide dismutase, STZ—streptozotocin.

## 6. Challenges in Translating Preclinical Findings and Other Limitations

As discussed in the previous sections, various strains, species, doses, and experimental models have been investigated in recent years, providing a growing body of evidence that probiotics can help mitigate oxidative stress and inflammatory markers in DM. These findings support the therapeutic potential of probiotics as an adjunctive strategy in DM and its complications. However, translating these promising results from preclinical research into clinical practice presents several challenges that must be acknowledged.

One of the primary limitations in generalizing the effects of probiotics is their strain-specificity. Different bacterial strains, even within the same species, may exert distinct biological effects due to variations in surface molecules, metabolic capabilities, and interaction with the host’s immune system. This makes it difficult to predict outcomes across different studies or populations. In Table 1, this information is clearly illustrated in the key findings associated with probiotic strains. Furthermore, the matrix in which probiotics are delivered (e.g., dairy products, capsules, or fermented foods) may influence their viability, absorption, and overall bioactivity, further complicating efforts to standardize treatment protocols [100].

Another important consideration is the dose–response relationship, which remains highly variable for many strains due to the different experimental models and objectives to be investigated. While some studies have shown benefits at relatively low CFU counts, others require higher doses to elicit measurable effects, and the optimal duration of treatment is also unclear ([74,75,76,77], in Table 1). These variables introduce heterogeneity that limits the comparability of results and underscores the need for rigorous, well-designed clinical trials. Addressing these translational challenges is crucial for determining the true clinical utility of probiotics in managing diabetes and its complications. Despite these critical limitations, we have gathered clinical evidence for the use of these probiotics in patients with DM.

Other limitations must also be highlighted. The therapeutic efficacy of probiotics in DM and its complications is not uniformly supported across the literature. In a 16-week intervention, T2D patients received a liquid *Lactobacillus* supplement (10^8^ CFU/mL), administered four times daily. The treatment failed to produce significant changes in either fasting blood glucose or HbA1c levels. Possible causes for treatment failure include reduced probiotic viability due to the inherent instability of liquid formulations under routine storage conditions, single-strain selection, and adherence variability [101].

Tipici et al. [102] reported that probiotic supplementation with *L. rhamnosus* GG (10 × 10^9^ CFU/day) in females with T2D showed no significant effects on blood glucose levels, HbA1c, fructosamine, HOMA-IR, or inflammatory parameters. The authors noted that while other studies have reported positive outcomes with probiotics, discrepancies may be attributed to strain selection, dosage, treatment duration, population characteristics, and sample size.

In a 6-week, randomized, double-blind, controlled clinical trial, 211 participants received whole-milk yogurt containing *L. acidophilus* LA5 and *B. lactis* BB12 (3 × 10^9^ CFU/mL)—either alone or associated with probiotic capsules at an equivalent dosage—or a placebo. None of the formulations significantly improved glycemic parameters (fasting glucose, insulin, and HbA1c). The authors propose that these null findings may reflect host genotype-dependent modulation of probiotic efficacy [103].

A meta-analysis of nine randomized controlled trials demonstrated that the consumption of probiotic yogurt did not significantly reduce serum HbA1c, fasting glucose, or insulin levels in patients with T2D and obesity. The authors suggest that the use of conventional yogurt as a control in some studies may have led to a misinterpretation of the results, since conventional yogurt brands potentially contain undefined probiotic bacteria at unknown concentrations, masking the effects of the treatment. Antioxidant effects were not evidenced [104].

In the four aforementioned studies that failed to show beneficial effects of probiotics in DM, the antioxidant properties of the treatments were not investigated. Our literature search only identified one study that evaluated the use of probiotics in DM and reported no significant impact on either glycemic control or oxidative stress biomarkers. A double-blind clinical trial was conducted, involving 34 patients with T2D. Six-week treatments with a pool of *Lactobacillus* (*L. acidophilus*, *L. bulgaricus*, *L. bifidum*, and *L. casei*) demonstrated discrete decreases in triglyceride levels, MDA, IL-6, and insulin resistance, which were not significant. The authors attributed the negative results to the small sample size and short intervention period [105].

Notably, some studies failed to demonstrate beneficial effects in glycemic control, diabetic neuropathy, and nephropathy, underscoring that the heterogeneity in outcomes was likely due to differences in viability, strain specificity, dosage, and treatment duration. Additionally, although we aimed to provide a comprehensive overview of the antioxidant potential of probiotics in DM and its main complications, we considered including studies evaluating their use in diabetic retinopathy to complete the triad of major DM complications. However, no eligible studies were found that addressed the antioxidant actions of specific bacterial strains in this context.

Finally, the frequent inclusion of multi-strain formulations and the lack of standardized clinical protocols hinder the attribution of specific outcomes to individual strains. These issues highlight the need for rigorously designed, strain-specific, placebo-controlled trials to determine the true therapeutic potential of probiotics in DM management.

## 7. Conclusions

Probiotics are emerging as a valuable adjuvant strategy for managing diabetes mellitus (DM) and its major complications, primarily by modulating oxidative stress and dampening systemic inflammation. Both preclinical and clinical studies highlight the hypoglycemic and antioxidant properties of specific strains—particularly from the genera *Lactobacillus* and *Bifidobacterium*—demonstrating improved glycemic control, enhanced endogenous antioxidant defenses, and the mitigation of complications such as DNR and DNP. These benefits are linked to reductions in lipid peroxidation, oxidative and nitrosative stress, and proinflammatory markers. In addition to these widely studied genera, strains from *Akkermansia*, *Saccharomyces*, and *Bacillus* have shown therapeutic potential, broadening the scope of probiotic interventions and encouraging the exploration of novel species and strains.

However, several challenges must be addressed before probiotics can be firmly established in clinical practice. These include strain-specific effects, optimal dosing, treatment duration, and long-term efficacy. The frequent use of multicomponent formulations further complicates the evaluation of probiotics’ isolated effects. To solidify their role as clinically viable antioxidant therapies for DM, future research must prioritize standardized protocols and large-scale, placebo-controlled trials evaluating probiotic monotherapy. Given the central role of oxidative stress in the pathophysiology of DM and its complications, continued investment in microbiome-targeted therapeutics remains a promising avenue.

## Abbreviations

AGEs	Advanced glycation end-products
CAT	Catalase
CFU	Colony-forming units
DM	Diabetes mellitus
DNP	Diabetic nephropathy
DNR	Diabetic neuropathy
GDM	Gestational diabetes mellitus
GLP-1	Glucagon-like peptide-1
GPx	Glutathione peroxidase
GSH	Glutathione
HFD	High-fat diet
HOMA-B	Homeostasis model assessment of β-cell function
HOMA-IR	Homeostasis model assessment of insulin resistance
hs-CRP	High-sensitivity C-reactive protein
MDA	Malondialdehyde
NF-κB	Nuclear factor kappa B
NO	Nitric oxide
Nrf2	Nuclear factor erythroid 2-related factor 2
PKC	Protein kinase C
ROS	Reactive oxygen species
SCFA	Short-chain fatty acids
STZ	Streptozotocin
SOD	Superoxide dismutase
TAC	Total antioxidant capacity
T1D	Type 1 diabetes
T2D	Type 2 diabetes
